# Immune-epigenetic-metabolic crosstalk: attempting to unravel the multidimensional mechanisms of immune evasion in endometriosis

**DOI:** 10.3389/fimmu.2026.1855106

**Published:** 2026-07-14

**Authors:** Wenyu Xu, Juan Du

**Affiliations:** School of Medical and Life Sciences, Chengdu University of Traditional Chinese Medicine, Chengdu, China

**Keywords:** endometriosis, epigenetics, immune evasion, immune microenvironment, metabolic reprogramming

## Abstract

The survival of ectopic endometrial lesions in endometriosis critically depends on their ability to evade immune recognition and clearance by the host, a process known as immune evasion. Recent studies suggest that this process is not driven by a single factor but rather results from a multidimensional, interactive regulatory network involving immune dysregulation, epigenetic remodeling, and metabolic reprogramming. The pathophysiology of endometriosis is characterized by reduced cytotoxic activity and functional exhaustion of effector immune cells, along with excessive activation and expansion of immunosuppressive cells, affecting both innate and adaptive immunity. The overexpression of immune checkpoint molecules further impairs immunological clearance. DNA methylation, histone modifications, and non-coding RNAs contribute to immune cell tolerance by stabilizing repressive transcription programs and silencing pro-inflammatory genes. These epigenetic mechanisms maintain a sustained immunosuppressive state through direct regulation of immune-related genes and hormone-metabolizing enzymes. Additionally, the hypoxic environment of ectopic lesions stimulates glycolysis, leading to the accumulation of metabolites such as lactate. Beyond directly impairing immune cell function, these metabolites act as signaling molecules or cofactors for epigenetic enzymes, thereby influencing chromatin states and gene expression, thus mechanistically and functionally linking metabolism to epigenetics. This review aims to systematically unravel this multidimensional mechanism, elucidate its synergistic role in disease progression, and potentially pave the way for future combined therapeutic strategies targeting this complex pathway. Such approaches offer a novel and promising means to overcome immune evasion and clinical resistance in endometriosis.

## 1 Introduction

Endometriosis (EMS) is a chronic disease characterized by the growth of endometrial-like tissue outside the uterine cavity (1). It affects up to 10% of women of reproductive age, although the disease is not strictly limited to this population. Endometriosis is found in 50–80% of women with pelvic pain and up to 50% of those with infertility. Globally, approximately 190 million people are estimated to be affected, with an economic burden exceeding $22 billion in the United States alone (2, 3).

Most women with endometriosis experience a range of symptoms, including dysmenorrhea, dyspareunia, constipation, chronic abdominal and pelvic pain, and infertility. In addition, the condition impairs the metabolism of the liver and adipose tissue, causes systemic inflammation, and modulates brain function, which results in increased pain perception as well as mood disturbances and anxiety disorders. Taken together, these symptoms significantly reduce patients’ quality of life (1, 3).

Multiple explanations of endometriosis pathophysiology exist, each attempting to explain how the tissue spreads throughout the abdominal cavity. Current theories agree on a framework that includes the interaction of dispersed endometrial cells with a supportive pelvic milieu. Sampson’s seminal theory, which postulates that retrograde menstruation deposits viable endometrial fragments into the peritoneal cavity, remains a cornerstone in our understanding of endometriosis pathogenesis. The amount of retrograde menstrual flow and the existence of obstructive abnormalities in the outflow tube lend credence to this notion (4). Although retrograde menstruation is a known and common phenomenon, only 10-15% of women develop endometriosis during their reproductive years. Thus, the overt onset of the disease requires subsequent mechanisms, with a key factor being an abnormal local immune-inflammatory microenvironment (5). Mesenchymal stem cells within the endometrium have been demonstrated to directly disseminate and develop into ectopic endometriotic lesions (6). Endometriosis may result from metaplastic alterations in peritoneal, pleural, and ovarian mesothelial cells, according to the coelomic metaplasia theory, which provides a reasonable explanation for extra-pelvic and uncommon clinical manifestations (7, 8). Additionally, other theories have been proposed, including hematogenous or lymphatic dissemination, serosal metaplasia, and Müllerian remnant induction. However, no single hypothesis can fully account for the diverse clinical manifestations (5, 9, 10).

Despite the high prevalence of endometriosis, 65% of affected women receive an incorrect initial diagnosis, largely due to suboptimal diagnostic methods. The gold standard for surgical diagnosis, usually achieved by diagnostic laparoscopy, is not perfect. It is not always reliable, has difficulties with early detection and therapy, and may ignore the illness (3, 11). Although endometriosis is widely recognized as an inflammatory, systemic, estrogen-dependent disease, the core pathological mechanism—how ectopic endometrial cells successfully survive and proliferate under host immune surveillance—remains incompletely elucidated. In recent years, extensive research has delved into its role in disease progression from immune, epigenetic, and metabolic perspectives: immune system dysfunction creates a supportive microenvironment for ectopic endometrial “colonization”; epigenetic alterations confer invasive, proliferative, and stem cell-like properties to these cells; and metabolic reprogramming, particularly abnormalities in pathways related to cellular energy supply, provides the material and energy foundation for sustained lesion growth (12–15). Nevertheless, the majority of current research is limited to linear, one-dimensional interpretations. It remains unclear how the immunological, metabolic, and epigenetic systems interact to form a precise, dynamic network that drives immune evasion in endometriosis.

Drawing on observations from cancer biology where epigenetic−metabolic crosstalk promotes immune evasion, we hypothesize that endometriotic lesions may actively initiate an “epigenetic−metabolic reprogramming” rather than passively adapting to their environment, potentially creating a vicious cycle of immune evasion and long-term lesion persistence (16). We explicitly note that cancer and endometriosis are distinct diseases, and similarities at the phenotypic level do not imply shared mechanisms. We tentatively term this the “Immune−Epigenetic−Metabolic Axis” as a working hypothesis requiring experimental validation. To test this hypothesis, several complementary approaches are suggested, including single-cell multi-omics on patient lesions, co-culture of endometriotic stromal cells with immune cells under metabolic stress, *in vivo* patient-derived xenograft models with pathway perturbations, and longitudinal analysis of recurrent lesions (17, 18). These approaches would help distinguish active reprogramming from passive adaptation and, if validated, could guide future synergistic therapies targeting immunological, epigenetic, and metabolic nodes. While we draw inspiration from cancer biology, we do not assume mechanistic equivalence. All cancer−derived concepts are explicitly identified as speculative hypotheses requiring direct validation in endometriosis.

Given the broad and conceptually multidimensional nature of this topic, spanning immunology, epigenetics and metabolism, a narrative synthesis approach was adopted to integrate findings from these complementary research domains. A systematic literature search was conducted in PubMed/MEDLINE, Web of Science, and the Cochrane Library for peer-reviewed articles published between 1986 and 2026. The search strategy was designed around three thematic blocks: immune evasion, epigenetic mechanisms, and metabolic alterations, combined with Boolean operators. Examples of the Boolean logic applied include: endometriosis AND (immune evasion OR immune cell), endometriosis AND (DNA methylation OR histone modification OR non-coding RNA), endometriosis AND (Warburg effect OR glycolysis OR lipid metabolism OR ferroptosis). The searches were limited to human studies and English-language publications. No restrictions were applied based on study design. Titles and abstracts were screened for relevance, and full texts of potentially eligible articles were then assessed for inclusion. We screened the literature according to the above method and propose a theoretical framework based on the selected studies.

## 2 Immune dysfunction

### 2.1 Recruitment and function of immune cells

**Figure 1:**
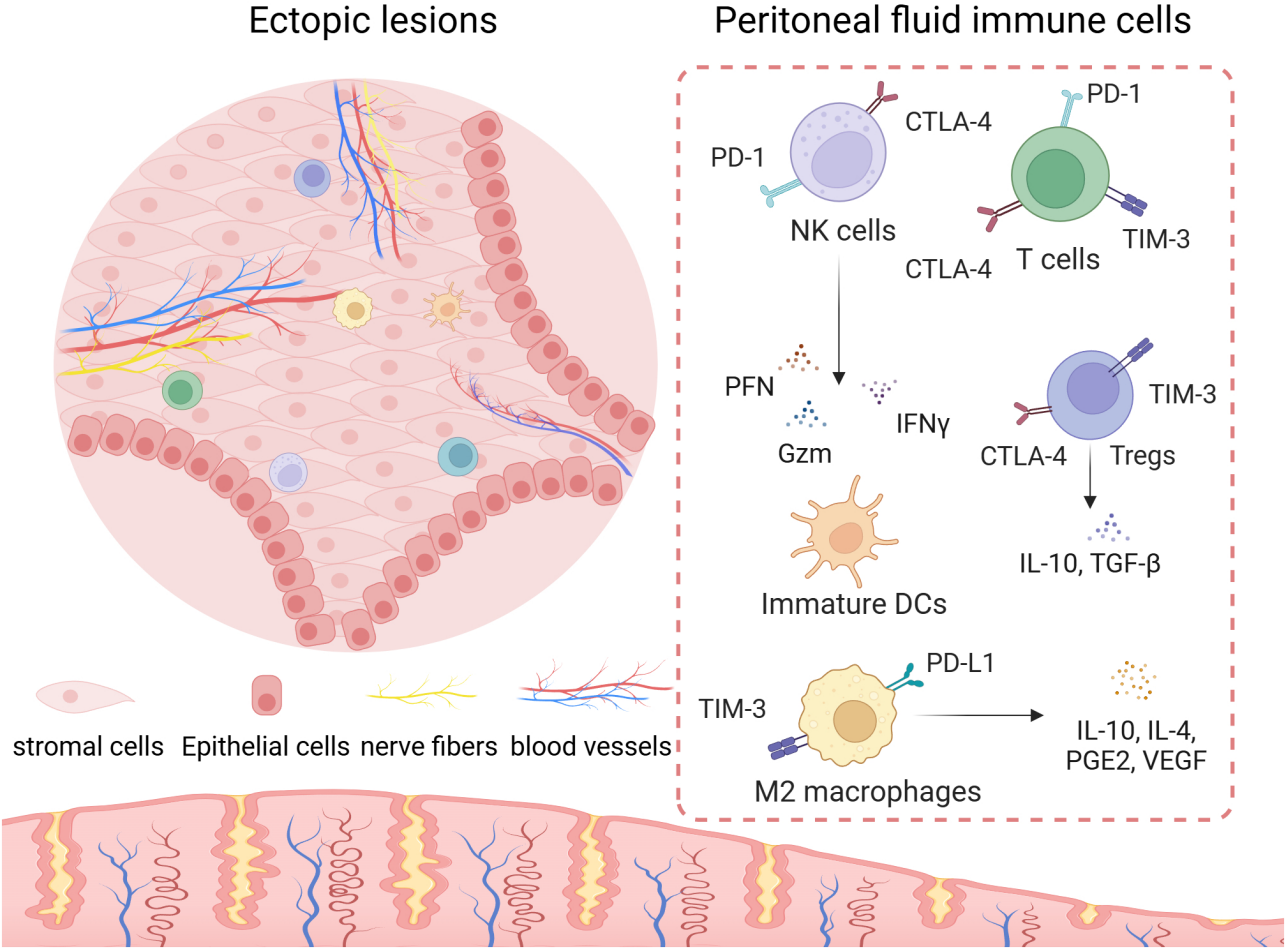
Ectopic lesion components and peritoneal fluid immune cells in endometriosis. Endometriotic lesions consist of epithelial cells, stromal cells, blood vessels, and nerve fibers. These components interact with immune cells (such as macrophages, natural killer cells, dendritic cells, B lymphocytes, and T lymphocytes) and multiple cytokines to collectively form an immune microenvironment. In endometriosis, the M2 macrophage phenotype is prevalent, accompanied by aberrant activity of NK cells and Treg cells. Created with BioRender.com.

The pathogenesis of endometriosis depends on immune regulation, as lesions thrive in an inflammatory, angiogenic, and endocrine microenvironment (19). It has been proposed that the immune system maintains peritoneal homeostasis, but definitive evidence that retrograde menstruation-derived endometrial cells are subject to classical immune surveillance is lacking (12). The primary immune effector network responsible for eliminating ectopic cells comprises macrophages, natural killer (NK) cells, and cytotoxic T lymphocytes (CTLs) (19). Previous studies have demonstrated that immune dysfunction is indeed present in endometriosis. When immune tolerance mechanisms become imbalanced, these immune cells collectively establish a locally immunosuppressive microenvironment within ectopic lesions, allowing ectopic endometrial cells to evade immune clearance (12). Immune evasion and persistent chronic inflammation are frequently observed in this milieu. The immunological microenvironment in EMS is pro-inflammatory in the early stages but shifts to an anti-inflammatory in the later stages (20). Despite immunosuppression, numerous activated immune cells infiltrate lesions and release proinflammatory cytokines, promoting tissue remodeling and inflammation (**Figure 1**).

Notably, endometriosis comprises several immunologically distinct phenotypes (peritoneal, ovarian, deep infiltrating, and adenomyosis) (21). The following discussion of immune cells focuses primarily on peritoneal and ovarian endometriosis. We acknowledge that the proposed axis may not apply to adenomyosis or deep infiltrating endometriosis without direct validation. NK cell heterogeneity also exists across compartments and patient subgroups (21). Therefore, this axis should be considered a peritoneal− and ovarian−dominant model requiring phenotype−stratified validation. Its purpose is to generate testable hypotheses, not to claim universal equivalence across all phenotypes.

#### 2.1.1 Macrophages

Macrophages play a key role in the development and inflammation of endometriotic lesions. Marina et al. found significantly elevated macrophage populations in peritoneal fluid and *in situ* endometrium of patients with endometriosis. Furthermore, these patients exhibited greater macrophage infiltration into endometriotic lesions than into healthy peritoneum (22). Macrophages can polarize into pro−inflammatory M1 or anti−inflammatory M2 phenotypes in response to environmental cues. In endometriosis, M1 macrophages secrete cytokines and chemokines that induce tissue damage and inhibit endometriotic cell growth. Conversely, M2 macrophages exert immunosuppressive effects that promote ectopic lesion survival, invasion, vascularization, and formation (23). M2 macrophages predominate in endometriotic lesions, where they promote lesion persistence through tissue fibrosis, angiogenesis, and extracellular matrix remodeling. Elevated M2 macrophage levels have been found in peritoneal fluid and endometrial tissue of endometriosis patients. These macrophages secrete immunosuppressive (IL-4, IL-10, TGF-β) and proangiogenic factors, which inhibit effector T cell and NK cell function while promoting regulatory T cell expansion, thereby facilitating immune evasion and survival of ectopic endometrial tissue (23, 24). Recent findings suggest that CD206^+^ macrophages, a hallmark of the M2 phenotype, contribute to endometriosis progression through the induction of local angiogenesis (25). Consequently, disease severity and progression are influenced by M2 polarization.

Endometriosis and certain cancers exhibit overlapping immunopathological features, including immune evasion and chronic inflammation. However, rather than assuming mechanistic equivalence, we use cancer literature in the following sections only as a source of testable hypotheses, clearly distinguishing speculation from endometriosis−specific evidence. Tumor-associated macrophages (TAMs) play a crucial role in the tumor microenvironment and are classified into pro-tumor (M2-like) and anti-tumor (M1-like) macrophages based on phenotype and function (26). Research shows that M2-like macrophages enhance tumor growth by promoting angiogenesis, fibrosis, tumor cell invasion, and proliferation. M2 TAMs attenuate cytotoxic effects and promote immunological tolerance by modulating other immune cells, including NK cells and CTLs (27). Endometrial implants trigger local immune responses at the lesion site, and evasion of immune surveillance facilitates implantation and lesion progression. Based on these parallels, it is tempting to speculate that M2−mediated immunosuppression and immune evasion could be shared features of endometriosis and cancer. However, critical differences exist (e.g., no malignant transformation, different tissue architecture and stromal cytokine signaling), and direct evidence in human endometriosis remains limited. Moreover, clinical translation of cancer-derived immunotherapies to endometriosis has been disappointing. For example, resveratrol reduced lesions in mouse models but failed in clinical trials (NCT02475564), and IDO inhibitors showed efficacy in melanoma but failed in a Phase 3 trial (ECHO−301/KEYNOTE−252). These examples illustrate the risk of uncritical extrapolation from cancer models (28). A key limitation of current animal models should be acknowledged. Rodent models cannot recapitulate deep infiltrating lesions, and no study has successfully modeled such lesions in rodents, despite their clinical severity. Moreover, immunodeficient nude mice used in heterologous models fail to replicate the inflammatory response central to human endometriosis (28). These constraints warrant caution when interpreting mechanistic findings derived solely or predominantly from rodent studies. Thus, while the TAM analogy offers testable hypotheses, any claims of shared mechanisms require direct validation in endometriosis-specific settings.

TET3 expression in macrophages is upregulated by factors including TGF−β1 and MCP1 in the peritoneal cavity of endometriosis patients, showing an eight−fold increase in peritoneal lesions compared to healthy endometrium. TET3 suppresses the miRNA regulator let−7, leading to increased production of the proinflammatory cytokines IL−6 and IL−1β. IL−6 activates the JAK/STAT3 pathway, promoting STAT3 recruitment to gene promoters that control inflammatory mediators, thereby driving macrophages toward a proinflammatory state. Thus, TET3 has been associated with enhanced STAT3 transcriptional activity in peritoneal macrophages, and may indirectly contribute to inflammatory responses via IL−6. However, causal directionality has not been demonstrated by longitudinal data (29). TET3 is dynamically regulated by the endometriotic microenvironment and influences macrophage function and survival. Macrophages overexpressing TET3 exhibit impaired immune surveillance, allowing ectopic endometrial cells to evade clearance and sustain lesion growth. However, this study had limitations, as it used PBMCs from healthy donors (not endometriosis patients) and only seven fixed endometriosis samples. Thus, while TET3 may contribute to endometriosis pathogenesis and deserves further investigation as a possible therapeutic target, current evidence is preliminary and requires validation in larger endometriosis−specific cohorts.

#### 2.1.2 Natural killer cells

NK Cells are renowned for their cytotoxic function, with their activation and suppression finely regulated by both activating and inhibitory receptors as well as cytokines. In endometriosis, NK cells recognize and eliminate ectopic endometrial cells. However, the cytotoxic capacity of NK cells is limited by the overexpression of inhibitory receptors and excessive activation. Thus, decreased cytotoxicity is the main feature of NK cells in patients with endometriosis (30).

NK cell activation is suppressed by the local peritoneal environment in endometriosis. Consistent with this, NK cells from affected patients exhibit an aberrant profile of activating and inhibitory receptors on their surface compared with those from healthy controls (31, 32). In endometriosis, peritoneal fluid levels of MICA and MICB are elevated. These proteins act as soluble ligands for the NK cell activating receptor NKG2D. Conversely, high MHC−I expression on target cells engages inhibitory receptors, thereby protecting them from NK cell attack (33). Furthermore, it has been suggested that tumors can evade immune surveillance and inhibit NK cell activity by increasing MICA and MICB expression (34, 35). Furthermore, Ricciarda et al. found a correlation between high expression of the inhibitory receptor CD94/NKG2A on peripheral blood NK cells from endometriosis patients and its ligand, human leukocyte antigen E, an interaction that suppresses NK cell cytotoxicity (36). Similarly, NK cell cytotoxicity is reduced upon the binding of their ligands, such as HLA-G, to the inhibitory receptors LILRB1 and LILRB2 expressed on NK cells (37). Animal models of endometriosis also exhibit reduced NK cell activity. In mice with induced endometriosis, both the ectopic lesions and peritoneal fluid suppress conventional NK cell function (38). Du et al. demonstrated that platelets protect endometriotic lesions from clearance by forming a “pseudo-self” envelope over them and downregulating the NKG2D ligand on these cells, thereby reducing NK cell cytotoxicity (39).

Reduced NK cell cytotoxicity has been reported in endometriosis (30). This reduction may impair immune surveillance and allow ectopic endometrial cells to persist, potentially contributing to immune evasion.

#### 2.1.3 Regulatory T cells

In endometriosis, the abnormal expansion and functional activation of Tregs are key drivers of immune evasion. Tregs infiltrating lesions strongly suppress effector immune responses through multiple mechanisms. The proportion of CD4^+^FOXP3^+^ regulatory T cells in the peritoneal fluid of women with endometriosis is significantly increased. Research shows that peripheral blood Treg populations are unaffected, whereas endometriosis patients have higher numbers of suppressive Tregs in peritoneal fluid than controls (40). Li et al. demonstrated that the supernatant from co-cultured human endometrial stromal cells (ESCs) and macrophages exerts multiple effects on Tregs. Specifically, it induces Treg differentiation via the AKT/STAT3 pathway, upregulates their expression of IL-10, TGF-β, and CD73, and suppresses their apoptosis by downregulating Fas and FasL. Together, these changes enhance Treg-mediated suppression of CD4^+^CD25^-^ T cells. IL-10 and TGF-β secreted by Tregs increased MMP2 expression and decreased TIMP1 expression, further stimulating ESC proliferation and invasion as well as ectopic lesion growth. As a result, TECK secreted by macrophages and ESCs increases the quantity and activity of Tregs in the ectopic environment, supporting high levels of ESC invasion and proliferation, as well as immunological tolerance in endometriosis (41). In a similar vein, CCL20 accelerates the development of endometriosis by promoting the migration of CD4^+^CD25highFOXP3^+^ Tregs (42). Increased macrophage activation, more effector T cells, and improved lesion development were also noted in animal models with reduced Tregs (40). These studies demonstrate that inflammatory mediators and antigen−presenting cells in the lesion microenvironment sustain Treg recruitment and activation, establishing a self−perpetuating immunosuppressive network. Multiple cell types and soluble factors collectively promote immune tolerance and persistent ectopic lesion adhesion and growth.

However, focusing solely on FOXP3+ Treg cells may be insufficient. Based on immunology and cancer literature, Treg cells have been subdivided into activated (aTregs), resting (rTregs), and non−suppressive (nTregs) subsets (43). ATregs are described as the primary inhibitory subset; rTregs can differentiate into aTregs upon stimulation; nTregs secrete IL−17 and promote B cell enrichment (44, 45). In endometriosis, direct evidence on these subsets remains very limited. One study reported that aTregs may impede dendritic cell maturation via IL−10, potentially encouraging immune evasion and lesion formation, and that nTregs may contribute to an immunosuppressive environment (46). However, these findings are preliminary, and most subset characterizations come from non−endometriosis studies. Thus, while the subset framework is informative, caution is needed when applying it to endometriosis. Future research should examine the Treg subset distribution and function specifically in patients with endometriosis.

#### 2.1.4 Dendritic cells

Dendritic cells (DCs) are specialized antigen−presenting cells that bridge innate and adaptive immunity, playing key roles in both activation and tolerance. DCs are classified into conventional (cDCs), plasmacytoid (pDCs), and monocyte−derived (moDCs) subsets, and can be immature, semi−mature, or mature based on phenotype and function. Immature DCs present self−antigens without co−stimulatory signals, promoting tolerance, whereas mature DCs present non−self antigens with appropriate signals, driving immunological clearance (47). The tumor microenvironment induces DCs to adopt a tolerance-inducing phenotype. These tolerance-inducing DCs not only fail to activate effector T cells effectively but also drive the differentiation and expansion of regulatory T cells, potentially directly inducing T cell dysfunction.

In addition to a lower percentage of mature DCs and a higher percentage of immature DCs in the peritoneal DC population, Zheng Qiaomei et al. found that endometriosis patients had higher peritoneal DC density (48). High peritoneal DC levels may promote endometriotic lesion formation, particularly in early disease stages, and DC maturation may significantly influence disease progression. Altered DC activity or phenotype may also contribute to ectopic implantation and endometrial growth. Suszczyk et al. reported significantly elevated proportions of Gal−9^+^ mDCs and pDCs in the peritoneal fluid of endometriosis patients, along with higher soluble Gal−9 and TIM−3 levels. They proposed that accumulation of Gal−9−expressing DCs together with elevated sTIM−3 and Gal−9 may represent a hallmark of immune dysregulation, exacerbating inflammation while establishing local immunosuppression (49). Yang Xu et al. reported that compared with normal endometrium, ectopic endometrium had fewer myeloid DCs, more CD11c^-^CD304^-^ DCs, and elevated HSD11B1 expression in both ectopic tissue and isolated myeloid DCs. They proposed that endometrial stromal cells overexpressing HSD11B1 secrete more cortisol, which suppresses DC maturation and cytokine production. Thus, HSD11B1 overexpression may contribute to endometriosis pathogenesis by preventing myeloid DC maturation (50).

Rather than depletion, DCs promote endometriosis through functional reprogramming from immunogenic activation to immune tolerance. By establishing a persistent immunosuppressive microenvironment, this shift ultimately facilitates immune evasion in ectopic lesions. Furthermore, strong evidence is required to clarify DCs’ role in endometriosis, given the complexity of their origin, phenotype, and function in the disease.

#### 2.1.5 Neutrophils, B cells, and myeloid−derived suppressor cells

Emerging evidence suggests that additional immune cell types participate in the immunosuppressive microenvironment of endometriosis. A study by Wilson et al. demonstrated that in ovarian endometrioma, infiltrating neutrophils acquire PD−L1 expression and suppress CD8^+^ T cell proliferation, representing a direct immune evasion mechanism (51). The same study also reported increased proangiogenic VEGFR1^+^ neutrophils and enhanced neutrophil extracellular trap formation in menstrual effluent and peritoneal fluid, which facilitates ectopic cell adhesion. Thus, neutrophils may contribute to both immune suppression and lesion establishment. B cells have also been implicated in endometriosis−associated immune dysregulation. Approximately 60% of infertile endometriosis patients test positive for autoantibodies, with p53 identified as the most common target (52). Furthermore, B cells secrete IL−10 and IL−35, cytokines that suppress inflammatory immune responses while paradoxically promoting endometrial cell proliferation. These findings suggest that humoral autoimmunity and B−cell−derived immunosuppressive cytokines may act in concert to support lesion survival. MDSCs expand rapidly following endometrial transplantation in mouse models, with monocytic MDSCs exhibiting higher immunosuppressive capacity (53). PGE_2_ concentrations positively correlate with MDSC percentages (54). Mendelian randomization further supports a causal association between CD14 on monocyte−derived MDSCs and endometriosis risk (IVW OR 1.047, p=0.021) (55). Together, these findings suggest that neutrophils, B cells, and MDSCs may contribute to the immunosuppressive niche that supports ectopic lesion progression.

### 2.2 Aberrant expression of immune checkpoint molecules

Based on immunology literature, immune checkpoints (ICPs) include both co−inhibitory and co−stimulatory molecules that tightly regulate T−cell function. Major co−inhibitory receptors are programmed cell death receptor-1 (PD-1), T cell membrane protein-3 (TIM-3), lymphocyte activation gene-3 (LAG-3), cytotoxic T lymphocyte-associated antigen-4 (CTLA-4), and TIGIT (56). Co−stimulatory molecules such as CD28, 4−1BB, OX40, ICOS, and GITR provide essential second signals for T−cell activation; among them, OX40L−OX40 signaling has been well characterized in inflammatory conditions (57, 58). In many cancers, tumor cells upregulate ICP ligands to evade immune attack, leading to T−cell exhaustion and impaired effector function. Disruption of the balance between co−stimulatory and co−inhibitory signals can also cause overt immune dysregulation; for instance, immune checkpoint inhibitor therapy may trigger autoimmune vasculitis, underscoring the critical role of ICPs in maintaining peripheral tolerance (58, 59).

Given this background, it is plausible that similar ICP−mediated mechanisms operate in endometriosis. By fostering immune tolerance toward ectopic endometrial implants and suppressing local and systemic immunity, ICPs may contribute to the early pathogenesis, lesion progression, and proliferation of endometriosis (56, 60).

#### 2.2.1 The PD-1/PD-L1 pathway in endometriosis

Recent studies suggest that the PD-1/PD-L1 pathway is implicated in the pathogenesis of endometriosis. As a major immune checkpoint, PD-1 exerts significant inhibitory effects in maintaining peripheral tolerance. However, dysregulated expression of PD-1 and its ligand PD-L1 may contribute to sustained T cell activation and the progression of inflammation and tissue damage. To investigate this, Walankiewicz et al. examined PD-1 and PD-L1 expression on T and B cells in peripheral blood from endometriosis patients and healthy women. They discovered that patients with advanced illness had greater frequencies of PD-1-positive T and B cells, suggesting that changes in the PD-1/PD-L1 axis may play a role in the etiology of endometriosis. The expression of PD-1 and PD-L1 on T and B cells may represent an immune system response to chronic antigen exposure in endometriosis patients. Consequently, disruption of the PD-1/PD-L1 axis may be associated with sustained T-cell activation, the development of autoimmunity, and the maintenance of inflammatory processes in these patients (61). Additionally, Wu et al. detected PD−1/PD−L1 expression in both normal and ectopic endometrium, with higher levels in EMS. PD−1/PD−L1 expression was also elevated in peripheral blood CD4^+^ and CD8^+^ T cells of EMS patients. Furthermore, 17β−estradiol treatment upregulated PD−L1 expression in eutopic epithelial cells from EMS patients. These findings suggest that the estrogen−regulated PD−1/PD−L1 pathway may contribute to immune dysfunction in endometriosis (62).

Interactions between DCs and T cells, mediated by the PD-1/PD-L1/PD-L2 pathway, are key regulators of immune tolerance and autoimmunity. Dorota et al. found that the peritoneal fluid of EMS patients contained higher levels of soluble PD-L1 and PD-L2 than plasma, as well as a higher percentage of mDCs and pDCs expressing PD-L1 or PD-L2 (63). Thus, altered PD-L1/PD-L2 expression on mDCs and pDCs in EMS patients may disrupt or even suppress appropriate T-cell activation, thereby promoting immunosuppression and supporting endometrial tissue implantation, proliferation, and growth within the peritoneal cavity. In a 2024 case-control study, Hosseinzadeh et al. compared the frequency of exhausted NK cells expressing PD−1 and/or TIM−3 in peripheral blood (PB) and peritoneal fluid (PF) of women with advanced endometriosis versus healthy controls. They found a higher proportion of PD−1^+^ NK cells in the PF of endometriosis patients, whereas the frequency of TIM−3^+^ or PD−1^+^ NK cells in PB did not differ significantly. These findings suggest that endometriosis is associated with a locally diminished NK cell response, which may resemble the exhausted T cell phenomenon (64). Flow cytometry was used to assess PD-1 and PD-L1 expression on T and B lymphocytes, and enzyme-linked immunosorbent assays were employed to measure their soluble forms in serum and ascites. Compared with controls, patients with endometriosis showed significantly higher expression of PD-1 and PD-L1 on T and B lymphocytes. Consistent with prior studies, high expression of both molecules was also associated with endometriosis stage. These findings implicate the PD-1/PD-L1 axis in immune dysregulation during endometriosis, suggesting that aberrant expression of these checkpoint molecules facilitates immune evasion by ectopic lesions, thereby promoting their survival and proliferation (60). PD−L1 expression on tumor cells and antigen−presenting cells induces T−cell exhaustion, DC dysfunction, and enhanced Treg differentiation, enabling cancer cells to evade immune control and resist CTL lysis. Similar immunosuppressive mechanisms may occur in endometriosis, allowing endometrial implants to escape host immune attack.

The immune evasion mechanism of endometriosis is closely linked to the PD−1/PD−L1 axis, which creates an immunosuppressive microenvironment through multi−level regulation. Inflammatory mediators in the ectopic lesion microenvironment induce infiltrating macrophages, dendritic cells, and ectopic endometrial cells to highly express PD−L1, while concurrently activating T and NK cells to upregulate PD−1. PD−1/PD−L1 expression is significantly elevated in both ectopic and eutopic endometrial tissues of patients, and peripheral blood levels correlate positively with disease stage, indicating systemic immune suppression. This pathway also promotes Treg cell development and the release of immunosuppressive factors such as TGF−β and IL−10, establishing immune tolerance. Moreover, 17β−estradiol upregulates PD−L1 expression in endometrial cells, enhancing PD−1/PD−L1−mediated immunosuppression and synergistically supporting lesion survival. These findings suggest that PD−1/PD−L1 may represent a therapeutic target in endometriosis, though further studies are needed to confirm its role in disease pathophysiology.

#### 2.2.2 The CTLA-4/CD80/CD86 pathway in endometriosis

Cytotoxic T-lymphocyte-associated antigen-4 (CTLA-4) exists in two forms: the membrane-bound protein CTLA-4 and the soluble form sCTLA-4, present in serum and peritoneal fluid (65). Notably, its soluble form (sCTLA-4) is connected to endometriosis-related infertility and other autoimmune disorders (66). CTLA-4 is a crucial inhibitory immunoregulatory molecule in the family of type I membrane receptors (67).

Membrane-bound CTLA-4 functions as a crucial regulatory factor in inhibiting lymphocyte activation by directly competing with CD80 and CD86, the same ligands that bind to the CD28 protein. By interacting with CD80/CD86 on DCs, CTLA-4 downregulates CD80/CD86 expression and upregulates tyrosine 2, 3-dioxygenase (IDO) expression, conferring DCs with immunological tolerance characteristics and transmitting inhibitory signals to antigen-presenting cells (68). In regulatory T cells, sustained CTLA-4 expression is essential for their immunosuppressive activity. CTLA-4 reduces T cell activation and proliferation, and inhibits IL-2 production (69). CTLA-4 serves a dual function in maintaining T cell tolerance. In regulatory T cells, CTLA-4 suppresses inappropriate activation of naive T cells, and its absence leads to abnormal activation and expansion of conventional T cells. Conversely, CTLA-4 expression on conventional T cells prevents abnormally activated T cells from infiltrating and damaging extra-lymphatic tissues, thereby limiting the accumulation of pathogenic autoreactive T cells in vital organs (70). Furthermore, studies suggest that elevated sCTLA-4 concentrations in the peritoneal fluid of EMS patients may confer tolerance-related functions analogous to those observed in malignant diseases. It is worth noting that soluble CTLA-4 exerts strong inhibitory effects on CD8^+^ T cells, leading to decreased proliferative capacity in mouse models (71).

Peritoneal sCTLA-4 plays an immunoregulatory role in the pathogenesis of endometriosis. Santoso et al. found that serum sCTLA-4 levels were significantly elevated in patients with advanced-stage endometriosis compared to those with early-stage endometriosis and controls. Furthermore, sCTLA-4 concentrations were higher in peritoneal fluid than in serum, and were also elevated in women with endometriosis-related infertility compared to their controls (66). Serum and peritoneal sCTLA−4 concentrations are positively correlated, suggesting the peritoneal cavity as a source of serum sCTLA−4. Abramiuk et al. reported that patients with more severe endometriosis had higher numbers of CTLA−4^+^ T cells than those with milder disease, based on analysis of CTLA−4 expression on T and B cells and its levels in serum and peritoneal fluid. However, sCTLA−4 concentrations in peritoneal fluid and plasma of the endometriosis group did not show previously reported associations with adhesions, infertility, or pelvic pain syndrome. Notably, the number of CTLA−4^+^ T lymphocytes negatively correlated with NK and NKT−like cell counts in infertile endometriosis patients, suggesting distinct mechanisms underlying infertility (72). Gueuvoghlanian−Silva et al. found higher proportions of CTLA−4^+^ Treg cells in the peritoneal fluid of endometriosis patients than in benign controls, and peripheral blood Treg proportions exceeded those in PF (73). Interestingly, Viganò et al. proposed two hypotheses regarding the autoimmune etiology of endometriosis: on the one hand, autoimmunity could serve as a foundation for endometriosis development independent of CTLA-4-related susceptibility; on the other hand, endometriosis may be unrelated to autoimmune etiology but may be associated with the progression of autoimmune responses. Viganò et al. found that the pathogenesis of endometriosis is not primarily associated with the development of CTLA-4-related autoimmune disease (74). Lerner et al. observed no difference in CTLA−4 + 49A/G polymorphism frequency between endometriosis patients (mild−to−moderate or severe) and healthy controls (75). Collectively, clinical studies suggest CTLA−4 contributes to chronic intraperitoneal inflammation, but genetic analyses do not support CTLA−4−related autoimmunity as a primary driver of endometriosis. Further research on CTLA−based immunoth Ectopic lesions actively initiate erapy, including adverse effects and novel strategies, is needed.

The CTLA−4 axis promotes immune evasion in endometriosis by enhancing Treg cell activity and suppressing antigen presentation, thereby supporting ectopic endometrial cell survival. In general immunology, CTLA−4 overexpression on Treg cells is known to reduce dendritic cell antigen presentation via immunosuppressive cytokines such as TGF−β and IL−10, thereby impairing effector T cell responses. Whether this mechanism operates in endometriosis remains to be directly demonstrated. Additionally, as a competitive receptor for B7 molecules, CTLA−4 inhibits CD28−mediated co−stimulation, forming a negative feedback loop that serves as a key node in maintaining immune tolerance.

#### 2.2.3 The TIM-3/Gal-9 pathway in endometriosis

Another crucial axis negatively regulating T cell activation and function involves the receptor TIM-3 and its ligand galectin-9 (Gal-9). Gal-9 is surface-expressed on eosinophils, T cells, Ma, or DCs, whereas TIM-3 is expressed on Th1, Th17, Mo/Ma, and DCs (76). Several studies show that when TIM-3 binds its ligand, Gal-9, it acts as a negative regulator, inducing Th1 cell exhaustion. This interaction also mediates peripheral immune tolerance, and blocking TIM-3 abolishes the development of Th1 cell tolerance (77). Furthermore, it impairs T cell proliferation and cytokine production (IL-2, IFN-γ, and TNF-α), ultimately inducing T cell death (78). In chronic viral infections, TIM-3 is highly expressed on both CD4^+^ and CD8^+^ T cells, leading to their exhaustion and weakened immune responses (79). Accumulating evidence suggests that blocking the TIM-3 signaling pathway restores the antigen-specific function of exhausted T cells. Preclinical studies have demonstrated the crucial role of TIM-3 in antitumor immunity, strongly supporting its potential as a target for immunotherapy.

Given TIM-3’s crucial role in immune system homeostasis, the TIM-3/Gal-9 pathway has recently drawn attention in endometriosis research, although studies on this pathway in endometriosis remain limited. First, Brubel et al. reported significantly elevated Gal-9 levels in both mild-to-moderate and severe endometriosis compared with healthy controls. Gal-9 ELISA showed excellent diagnostic value, outperforming other biomarkers. Elevated Gal-9 expression was also found in peritoneal fluid cells of endometriosis patients, and increased serum Gal-9 levels were observed in various benign gynecological conditions associated with pelvic pain or infertility (80). Meggyes et al. analyzed TIM−3 and Gal−9 expression on lymphocytes from PB and PF in women with and without endometriosis. They found significantly reduced TIM−3 labeling on all T and NK cell subsets in PB of the patient group, whereas Gal−9 positivity was elevated on PB CD4^+^ T cells and Tregs. In PF, both TIM−3 expression on CD4^+^ T cells and Gal−9 labeling on all T and NK cell subsets were markedly increased. The authors proposed that endometriosis may involve a TIM−3/Gal−9−dependent regulatory failure, leading to compromised immune surveillance and enhanced survival of ectopic lesions (81).

Notably, Tian et al. found significantly higher levels of TIM-3 mRNA and protein expression in ectopic endometrial tissue compared to normal endometrial tissue. They tested TIM-3’s role in regulating proliferation via the brain-derived neurotrophic factor-mediated PI3K/AKT axis by transfecting ESCs with plasmids and administering inhibitors in an endometriosis rat model. Ultimately, they concluded that TIM-3 may influence the proliferation of ESCs and the development of endometriotic lesions via the brain-derived neurotrophic factor-mediated PI3K/AKT pathway (82). Reis et al. found that patients with endometriosis exhibited a significantly reduced percentage of TIM-3-expressing CD56^+^ T cells in their PB. Furthermore, women with intestinal endometriosis and those newly diagnosed with the disease showed a significantly reduced percentage of TIM-3-expressing CD56^+^ T cells. However, further studies are needed to determine whether this contributes to endometriosis progression by amplifying autoreactive T cells and exacerbating inflammation (83). In addition to evaluating soluble Gal-9 and TIM-3 concentrations, Suszczyk et al. evaluated Gal-9 expression on mDCs and pDCs in PB and PF from EMS patients and healthy participants. They discovered that the PF of EMS patients had much greater quantities of soluble Gal-9 and TIM-3 than the peripheral circulation, as well as significantly higher proportions of mDCs-Gal-9^+^ and pDCs-Gal-9^+^. Therefore, Suszczyk et al. proposed that elevated Gal-9 expression on mDCs and pDCs in PF, along with high sTIM-3/Gal-9 production in the peritoneal cavity, may represent key immunoregulatory features in EMS patients, potentially exacerbating inflammation and local immune suppression (49).

Gal−9 is elevated in the peritoneal fluid/lesions of endometriosis patients. Binding TIM−3, which suppresses CD4^+^ Th1/cytotoxic T cells and induces apoptosis. Persistent TIM−3 on exhausted T cells/Tregs enhances immunosuppression and reduces NK cytotoxicity, impairing immune surveillance and promoting lesion survival. TIM−3 also mediates antitumor immunity, expressed on tumor−infiltrating T cells, macrophages/DCs, and promotes invasion/migration/growth in ovarian, cervical, prostate, and liver cancers. TIM−3 blockade induces antitumor responses and may synergize with other checkpoint inhibitors (84).

## 3 Epigenetic dysregulation in endometriosis

Recent studies on epigenetics have shed new light on the pathophysiology of EMS. In addition to affecting angiogenesis, apoptosis, and cell proliferation, aberrant control of DNA methylation, histone modifications, and non-coding RNAs also contributes to immune evasion by altering the expression of immune-related genes. Ectopic endometrial cells and the local immunological milieu are carefully programmed by this, allowing them to successfully evade immune monitoring by deftly “disguising” themselves and suppressing immune responses. This provides a new perspective for investigating the disease’s pathophysiology and developing innovative treatment approaches (85).

### 3.1 DNA methylation

As endometriosis is a persistent disease characterized by significant gene dysregulation, some form of cellular memory must constitute the unique cellular identity of endometrial cells. Epigenetic regulation, particularly through DNA methylation, serves as a flexible and stable mechanism for maintaining this cellular memory (86).

DNA methylation involves the addition of a methyl group to the C5 position of cytosine, forming 5−methylcytosine at CpG dinucleotides, catalyzed by DNA methyltransferases (DNMT1, DNMT3A, DNMT3B). Methylation in gene promoter regions represses transcription by recruiting methyl−binding proteins and blocking transcription factor binding. Through DNMT1 activity during replication, DNA methylation serves as an epigenetic memory system that stably maintains gene repression within a cell population (87).

Several studies have examined DNMT expression in endometriosis. Wu et al. examined DNMT expression in epithelial cells from ectopic endometrium, eutopic endometrium of endometriosis patients, and normal endometrium from healthy controls. Compared with healthy controls, ectopic lesions showed significantly higher DNMT1 expression, whereas DNMT3A and DNMT3B levels did not differ consistently. In contrast, eutopic endometrium from patients exhibited elevated DNMT3A alone relative to controls. Positive correlations among DNMT1, DNMT3A, and DNMT3B expression were observed in ectopic tissues (88). Peng et al. reported that hypoxia in endometriotic lesions reduces DNMT1 expression and PTGIS promoter methylation, increasing PTGIS transcription and PGI_2_ production. Elevated PGI_2_ supports early lesion survival and may drive angiogenesis/inflammation. Excess PGI_2_, via PTGIR, promotes CD16^+^ NK cell differentiation through CD16, reducing NK cytotoxicity and fostering peritoneal immunosuppression (89). These seemingly contradictory findings may reflect context−dependent regulation. Wu et al. examined epithelial cells under unspecified oxygen conditions, whereas Peng et al. studied whole lesions under hypoxia. DNMT1 expression may vary by cell type, lesion stage, and oxygen tension. Thus, rather than being inconsistent, these studies may capture different facets of DNMT1 regulation in endometriosis. This possibility should be addressed in future studies.

However, altered expression of DNMT enzymes alone does not directly demonstrate that DNA methylation changes contribute to pathogenesis. Direct evidence of DNA methylation levels and specific methylated targets in endometriosis has been reported. Benkhalifa et al. found that serum circulating cell−free DNA (Cf−DNA) levels were 3.9−fold higher in endometriosis patients than in healthy controls. They also observed elevated Cf−DNA carrying epigenetic methylation differences in four genes (RRP1, DIPC2, USP1, DNMT1) in patients with pelvic pain and endometriosis. Hypomethylation of these genes may abnormally activate inflammation (87). Using genome−wide methylation arrays, Dyson et al. identified 42, 248 differentially methylated CpGs in endometriosis compared to healthy endometrial cells, with significant differences mapped to 403 genes, including the HOX gene clusters, nuclear receptor genes, and the GATA family of transcription factors. Specifically, GATA2 was hypermethylated and repressed in endometriotic cells, while GATA6 was hypomethylated and overexpressed, potently blocking hormone sensitivity (90). A recent systematic review of 70 studies demonstrated that endometriosis is associated with DNA methylation modifications at specific genes involved in key signaling pathways, including PI3K−Akt, Wnt, and MAPK, as well as processes such as hormone response, immunity, and cell adhesion (91).

Collectively, altered DNMT expression, specific gene promoter methylation changes, and genome−wide methylation differences suggest that DNA methylation may contribute to endometriosis pathogenesis. However, it remains unclear whether these epigenetic modifications are causes or consequences of the disease (92). While DNA methylation−regulated processes may each contribute, individually they are insufficient to explain disease progression. Although the precise epigenetic drivers are unknown, accumulating evidence suggests that endometriosis is associated with polyepigenetic alterations affecting multiple key pathways.

### 3.2 Histone modifications

Histones are nucleoproteins that help DNA condense into chromatin by compressing it into the structural units of nucleosomes. The core of each nucleosome, which is the basic building block of chromatin, is a histone octamer made up of two units of each of the H2A, H2B, H3, and H4 histones (93). Acetylation, methylation, and ubiquitination are examples of post-translational modifications of histones that can change chromatin structure and their roles in transcription, replication, and repair processes. These alterations involve key enzymes including histone deacetylases (HDACs), histone acetyltransferases (HATs), histone methyltransferases (HMTs), and demethylases (93). HDACs regulate gene expression by deacetylating lysine residues on histones, which alters chromatin structure and leads to transcriptional repression. Through this mechanism, HDACs control cell cycle progression, proliferation, and differentiation. Like promoter methylation, histone deacetylation typically results in gene silencing, positioning HDACs as transcriptional repressors in cancer and other diseases (94).

Xiaomeng et al. examined histone H3/H4 acetylation and H3K4/H3K9 methylation in normal and ectopic endometrium from endometriosis patients, along with mRNA levels of related genes. They found low histone H4 acetylation in both tissue types, but no difference in global H3 acetylation between patients and controls. Low H3K4 methylation was detected in ectopic endometrium, whereas low H3K9 methylation was observed in normal endometrium. Additionally, patients had higher HDAC2 mRNA in normal endometrium and lower HDAC1 mRNA in ectopic lesions (95). Colón−Díaz et al. found that HDAC1 and HDAC2 expression varies by lesion location. In their tissue microarray that included four umbilical/skin endometriosis samples, HDAC2 was strongly expressed in all four skin lesions and in 82% of eutopic endometrial stroma from endometriosis patients, whereas HDAC1 was more frequent in gastrointestinal, ovarian, and skin lesions (96). Given the small number of skin samples (only four), these results should be interpreted with caution. Another study found significantly reduced H3 acetylation in endometriotic lesions compared with control endometrium, whereas H4 acetylation showed no significant change, suggesting inter−patient variability. Furthermore, lesion tissues exhibited reduced H3K9ac and H4K16ac relative to normal endometrium from both patients and controls. Compared with control endometrium, patient lesions also showed hypermethylation at H3K4, H3K9, and H3K27, indicating extensive heterochromatin suppression (97).

In healthy women, SIRT1 (a Class III NAD^+^−dependent deacetylase) expression in the endometrium is restricted to menstruation. However, SIRT1 is markedly overexpressed in the stroma and epithelium of endometriosis patients. In mouse uteri, aberrant SIRT1 expression leads to subfertility due to progesterone resistance, defective decidualization, and implantation failure. Thus, SIRT1 overexpression in endometriotic lesions is thought to promote disease progression (98). In addition to SIRT1, classical histone deacetylases (Class I) have also been implicated in endometriosis. Mai et al. investigated HDAC2 using a syngeneic mouse model established in NOD/SCID immunodeficient mice (intraperitoneal injection of donor uterine tissue fragments). They found that HDAC2 is upregulated while HNF4A and ARID1A are downregulated in endometriotic tissues; silencing HDAC2 correspondingly suppressed HNF4A expression. HNF4A was shown to bind the ARID1A promoter and activate ARID1A, thereby reducing human endometrial stromal cell (hEM15A) viability and invasiveness, leading to reduced lesions, decreased cell proliferation, and enhanced apoptosis (99). To assess the therapeutic potential of histone deacetylase inhibitors (HDACIs), Chadchan et al. used a syngeneic immune−competent mouse model (endometrial fragments transplanted intraperitoneally). High HDAC1 expression was observed in ectopic lesions by anti−HDAC1 staining. Intraperitoneal injection of n−butyrate, curcumin A, sulfonylurea hydroxyantipyrine, or entinostat resulted in fewer and smaller lesions, characterized by thinner stroma and epithelium (100).

The above studies suggest that dysregulated HDAC expression leads to a disease phenotype characterized by increased cell proliferation and invasion, while inducing cell cycle arrest and promoting apoptosis, thereby creating an immune-tolerant microenvironment conducive to the survival and progression of ectopic lesions. As a key epigenetic mechanism, histone modifications play a central role in the immune evasion process in endometriosis by dynamically regulating chromatin states and gene transcription.

### 3.3 Non-coding RNAs

Non−coding RNAs (ncRNAs) regulate genomic stability, transcription, post−translational modifications, and lipid interactions. They are classified by size into short RNAs, long non−coding RNAs (lncRNAs), and circular RNAs (circRNAs). Short RNAs include microRNAs, transfer RNAs, tRNA−derived fragments, and PIWI−interacting RNAs (101). Notably, miRNAs and lncRNAs act as key epigenetic regulators, fine−tuning immune−related gene expression and contributing to immune evasion in endometriosis. Dysregulated ncRNAs disrupt homeostasis and drive disease. Through autophagy modulation, aberrant ncRNAs promote cancer progression, metastasis, drug resistance, and stem cell proliferation (101).

MiRNAs regulate cell differentiation, proliferation, migration, and inflammation in EMS. Differentially expressed miRNAs have been identified in EMS, suggesting their potential as diagnostic biomarkers (102). Several miRNAs modulate inflammatory mediators via the NF-κB pathway: miR-199a suppresses NF-κB activation by downregulating IKKβ (103), and miR-16 inhibits ESC migration and invasion by blocking this pathway (104). However, neither miR-199a nor miR-16 is specific to endometriosis, as both have been implicated in other diseases such as cancer and inflammation. Thus, their roles in endometriosis should be considered part of broader regulatory mechanisms rather than disease−specific pathways. NF-κB target genes regulate multiple biological processes, such as cell proliferation, adhesion, anti-apoptosis, angiogenesis, oxidative stress, inflammation, and invasion. The NF-κB signaling network plays a critical role in the development and maintenance of endometriotic lesions. By triggering the MAPK pathway, the non-coding RNA miR-887-5p increases IL-10 release and M2 macrophage polarization. The anti-inflammatory cytokine IL-10 is essential for reducing inflammatory and autoimmune reactions. Ascites fluid and the culture media of ascites macrophages from endometriosis patients have been shown to contain significantly higher quantities of IL-10 protein (105). Studies show that miR-34c-5p and miR-142-3p contribute to macrophage phagocytic dysfunction and perform roles in inflammatory responses (106). Beyond intracellular miRNAs, exosomes from endometriosis patients deliver miR−210−3p, which suppresses JNK signaling and inhibits M1 macrophage polarization, while decreased exosomal miR−196a−5p activates the Hippo pathway to drive M2 polarization (107). Several studies suggest that NK cell dysfunction in endometriosis patients allows peritoneal reflux fragments to evade immune surveillance. miRNAs modulate NK cytotoxicity: miR−20a enhances it by triggering perforin, and miR−182 increases NKG2D/NKG2A−mediated killing in hepatocellular carcinoma (108). Moreover, when NK cells are co−cultured with autophagy−deficient endometrial cells, reduced miR−1185−1−3p in NK cells correlates with elevated COX−2/PGE2 and NK cells are low in granzyme, perforin, and IFN−γ. Autophagy inhibition induces FCGR3−positive NK cells, reduces cytotoxicity, upregulates IL8/IL23A, and downregulates HCK via STAT3 inactivation, thereby accelerating ESC proliferation and endometriosis progression (109).

Dysregulated lncRNAs are implicated in endometriosis pathogenesis. HOTAIR, a regulator of HOX genes, is critical for endometrial homeostasis, and endometriosis patients show increased likelihood of HOTAIR SNP variants (110). Zhang et al. reported that endometriotic lesions have lower miR−761 and higher HOTAIR expression (111). Mechanistically, HOTAIR acts as a ceRNA that sponges miR−761, thereby derepressing HDAC1. Consistent with this, miR−761 mimics inhibit HDAC1, whereas vesicular HOTAIR upregulates HDAC1. Vesicular HOTAIR suppresses ESC apoptosis and promotes migration, invasion, and proliferation; these effects are reversed by miR−761 upregulation or HDAC1 silencing. The HOTAIR/miR−761/HDAC1 axis drives inflammation via STAT3−dependent cytokines, and the STAT3 inhibitor stattic abrogates HOTAIR’s effects (111). Other lncRNAs are also dysregulated in endometriosis, as shown by Cui et al., who identified 86 differentially expressed lncRNAs in ovarian lesions (112).

In endometriosis, lncRNAs act as miRNA sponges. Decreased H19 lncRNA levels increase let-7 miRNA activity, which suppresses IGF1R expression and reduces endometrial stromal cell proliferation (113). According to another study, H19 reduces miR-124-3p via sponge-mediated inhibition, thereby increasing ITGB3 expression and controlling the ability of ectopic endometrial cells to proliferate and invade (114). CDKN2B-AS1 is another lncRNA shown to have a sponge function. In an *in vitro* model of ovarian endometriosis, it binds to miR-424-5p to control AKT3 expression (115). LINC01116 promotes proliferation and migration of endometrial stromal cells by sponging miR-9-5p to target FOXP1, thereby accelerating the formation and progression of endometriotic lesions (116). Additionally, other lncRNAs also regulate the proliferation and migration of endometrial stromal cells in patients by sponging miRNAs, participating in the development of endometriosis.

In general, ncRNAs regulate endometrial cell migration, invasion, proliferation, apoptosis, and inflammation, potentially affecting lesion implantation (117). They also modulate immune cells including NK cells and macrophages. Impaired macrophage and NK cell function compromises immune surveillance, allowing ectopic endometrial cells to evade clearance and proliferate. Inflammatory pathways such as NF-κB and JAK-STAT are activated by epigenetic changes, which exacerbate aberrant cytokine secretion, promote damage-associated molecular patterns release, and sustain chronic inflammation (117). This establishes a vicious cycle of immune evasion, lesion progression, and inflammation exacerbation.

DNA methylation, histone modifications, and non-coding RNAs are epigenetic mechanisms linked to the development and progression of endometriosis. Together, these mechanisms form a complex regulatory network that modulates hormone sensitivity, cell proliferation, apoptosis, and inflammation (**Table 1**). Epigenetic dysregulation of genes governing steroid hormone production/signaling, immune modulation, and endometrial cell identity and function has been implicated in the pathophysiology of endometriosis and its related infertility (118, 119). Although previous studies have shown links between immune evasion by endometrial cells and epigenetic regulation, more research is needed to determine the precise pathways. For example, it is still unknown if DNA methylation is a cause or an effect of the illness. It remains unclear how environmental factors influence immune evasion via epigenetic pathways, and the therapeutic potential of targeting non-coding RNAs has not been fully investigated (92).

**Table 1:** The role of epigenetic regulation in immune evasion of endometriosis.

Mechanism	Key targets/pathways	Immune evasion effect	Epigenetic crosstalk	References
DNA methylation	Hypomethylation: HLA−G, PD−L1; Hypermethylation: Fas, TNF−αR, MICA/B	Suppresses NK/CTL cytotoxicity; reduces apoptosis; impairs NK recognition	Methylated CpG islands recruit HDAC/HMT complexes (H3K9me3/H3K27me3); hypermethylation of MIR124 promotes M2 polarization	(88, 89, 118, 119)
Histone modification	HDAC: HLA−DR; H3K4me3: TGF−β, IL−10; H3K27me3: NKG2D	Impairs antigen presentation; promotes Treg differentiation; reduces NK cell recognition	Histone marks guide DNA methylation (H3K4me3 inhibits, H3K27me3 coexists with hypermethylation); lncRNAs recruit histone modifiers	(94, 118, 119)
Non-coding RNA	miR−21, miR−155; MALAT1, H19	Suppresses CTL/NK/macrophage function; promotes invasion and angiogenesis	miRNAs target DNMTs and EZH2; miRNA expression regulated by DNA methylation/histone modifications	(103, 106, 114, 118)

## 4 Metabolic reprogramming

Metabolic reprogramming has recently emerged as a key link between cellular metabolism and immune function in endometriosis immune evasion. Cells actively modify fundamental metabolic pathways—including glucose, lipid, and amino acid metabolism—to support growth, survival, and function. In endometriosis, ectopic lesions reprogram both their own energy metabolism and that of the microenvironment, creating an immunosuppressive milieu that enables evasion of immune recognition and attack. This allows ectopic endometrial tissue to escape immune surveillance and persist, providing a crucial pathological basis for disease progression (85).

### 4.1 Glycolysis and lactic acid accumulation

In cancer biology, the Warburg effect describes the preferential use of glycolysis even under aerobic conditions, a metabolic switch also observed in endometriosis (120). A hallmark of metabolic reprogramming is altered glucose metabolism. Glycolysis and mitochondrial biogenesis markers (GLUT1, MCT2, DRP1) are elevated in the blood and peritoneal fluid of adolescents with peritoneal endometriosis, indicating systemic metabolic alterations from early-stage disease. Simultaneously, there are notable changes in the cellular expression levels of mitochondrial biogenesis markers and glycolysis markers (HEX2, GLUT1, PDK1, MCT1, MCT2 and TGF-β) (121). However, as these studies primarily measured expression levels, which do not always directly correlate with enzymatic activity, functional assays are needed to confirm increased glycolytic flux.

Due to insufficient blood flow, endometrial tissue that is implanted ectopically frequently experiences hypoxia. Hypoxia is well known to induce angiogenesis in endometriosis, but the focus here is on its direct effects on glycolysis (122). As a fundamental regulator in hypoxic microenvironments, hypoxia-inducible factor-1α (HIF-1α) markedly increases the expression of important glycolytic enzymes, such as pyruvate kinase M2 (PKM2), phosphofructokinase 1 (PFK1), and hexokinase 2 (HK2), directly promoting glycolytic pathway activation (85). Increased expression of lactate dehydrogenase A (LDHA), which converts pyruvate to lactate, is another marker of active glycolysis in adult endometriosis patients (123). Endometriotic epithelial (11Z) and endometrial stromal (ESC) cell lines derived from endometriosis lesions have been shown to exhibit elevated expression of phosphofructokinase 6-phosphate/fructose-2, 6-bisphosphatase 3 (PFKFB3), which promotes disease progression (124). HK2 and PKM2 mRNA and protein levels are significantly higher in ectopic versus eutopic endometrium and positively correlate with lesion invasion depth. HIF-1α induces epithelial-mesenchymal transition in endometrial cells, accelerating endometriosis progression (125).

Glycolysis produces large amounts of lactate, and elevated peritoneal lactate in endometriosis patients promotes angiogenesis, immune evasion, and cell invasion (125). TGF-β1 levels are correlated with the concentration of lactic acid in peritoneal fluid. Mesothelial cells exposed to TGF-β1 produce more lactic acid, have higher levels of HIF1A mRNA and protein, and have higher mRNA concentrations of genes associated with glycolysis (LDHA, PDK1, SLC2A1) (123). Thus, at lesion sites, increased TGF-β, HIF-1α, and lactate accumulation may synergistically stimulate cell invasion and proliferation while promoting local immune tolerance (124). In endometrial ovarian cysts, High Mobility Group Box 1 (HMGB1) contributes to glycolysis, fosters the development of an immunosuppressive milieu, and intensifies persistent inflammation (126). Sun et al. reported that CHIP negatively regulates endometriosis progression by reducing HMGB1 expression. CHIP binds HMGB1, promoting its ubiquitination and degradation, thereby blocking aerobic glycolysis and inhibiting cell migration/proliferation (127). CHIP also suppresses glycolytic activity and invasive migration of ectopic endometrial cells by ubiquitinating the key glycolytic enzyme PFKFB4 and promoting its degradation, thereby precisely regulating glycolysis intensity.

### 4.2 Metabolism disorders of other substances

In addition to glycolysis, the metabolism of other substances also plays a certain role in the development of EMS. Extensive metabolic reprogramming and cancer-like changes have been observed in EMS, similar to the process of tumorigenesis.

Lipid metabolism plays a crucial role in cell membrane synthesis, signal transduction, and energy storage (128). Emerging evidence suggests that endometriosis involves alterations in lipid−related processes, although direct metabolomic confirmation has only recently been reported (129). Using transcriptomic analysis, Tu et al. identified 17 differentially expressed genes involved in fatty acid metabolism in endometriotic lesions, including PTGS2, CYP2C9, HSDL2, HSD17B3, ACSL4, and CYP2C18 (130). Among these, ACSL4 correlated positively with effector memory CD8^+^ T cells, whereas HSDL2 showed a negative correlation with activated CD8^+^ T cells. These genes are implicated in arachidonic acid metabolism, apoptosis, and immune cell interactions (130). However, differential gene expression alone does not directly demonstrate altered metabolic flux; validation using lipidomic or metabolomic approaches is required. Su et al. identified four gut microbiota genera associated with endometriosis risk using a bidirectional Mendelian randomization approach. Olsenella’s impact on endometriosis may be mediated by triglycerides, according to additional research (131). Yang et al. reported elevated total cholesterol levels in endometriosis patients compared with controls (132). Zhou et al. identified a bidirectional causal relationship between endometriosis and dyslipidemia, and showed that genetically predicted levels of PCSK9, APOB, and ANGPTL3 correlate with early-stage endometriosis risk, suggesting that lipid-lowering drugs targeting these molecules may have therapeutic potential (133). These findings, derived from blood samples, represent systemic associations and do not directly reflect local metabolic changes in ectopic lesions. Additionally, patients with EMS had considerably higher quantities of PGE2 and LTB4 in their peritoneal fluid, which is positively correlated with pain levels and lesion recurrence rates (134). This suggests local eicosanoid metabolism and inflammation, but does not inform on global lipid biosynthesis within lesions. In a comprehensive lipidomics study, Dai et al. performed LC−MS analysis of follicular fluid from endometriosis−associated infertility patients and identified elevated phosphatidylinositol (PI 16:0/18:2) and reduced lysophosphatidylinositol (LPI) species, which correlated with oocyte outcomes. LPI attenuated oxidative stress and apoptosis in granulosa cells via MAPK−ERK1/2 signaling, providing direct lipidomic evidence linking aberrant lipid metabolism to reduced fertility in endometriosis (129). Transcriptomics and lipid profiling suggest altered metabolism in endometriosis, but lesional tissue validation is lacking. Thus, current findings are hypothesis−generating and require larger standardized cohorts.

Glutamine and tryptophan metabolism are prominent amino acid anomalies in endometriosis. In peripheral blood, patients show significantly higher glutamine and lower tryptophan levels compared to controls (135). In ectopic lesions, expression of the glutamine transporter ASCT2 and glutaminase (GLS) is markedly increased, facilitating glutamine uptake and breakdown. GLS converts glutamine to glutamate, which is then converted to α−ketoglutarate (α−KG) by glutamate dehydrogenase (GDH). α−KG provides carbon sources for fatty acid and nucleotide synthesis and enters the TCA cycle, supporting the rapid growth of ectopic endometrial cells (136). Wang et al. demonstrated that the IGF2BP3/UCA1/c-MYC/GLS1 axis promotes glutamine metabolism, thereby enhancing endometriotic cell migration and proliferation. In this axis, IGF2BP3, an RNA-binding protein, interacts with UCA1 to stabilize GLS1 mRNA (137). Ectopic cells and infiltrating immune cells highly express indoleamine 2, 3−dioxygenase (IDO), which converts tryptophan to kynurenine. Thus, endometriosis is associated with alterations in tryptophan and glutamine metabolism, although the functional implications require further investigation (135).

Iron is an essential cofactor for enzymes involved in glycolysis, oxidative phosphorylation, and lipid biosynthesis. Ectopic endometrial tissue exhibits significant iron overload. On one hand, retrograde menstruation causes local bleeding, releasing hemoglobin that is converted to free iron by heme oxygenase−1. On the other hand, ectopic cells upregulate transferrin receptor 1, enhancing iron uptake (138). Skarzynska et al. proposed that assessing iron metabolism in women with endometriosis may serve as a biomarker for the disease and its progression. They found that both lactoferrin and transferrin levels in the peritoneal fluid of patients with endometriosis were lower than in plasma, whereas ferritin and iron content showed the opposite trend. In non-endometriosis patients, peritoneal lactoferrin levels positively correlated with plasma lactoferrin, iron, and transferrin concentrations, and the ferritin ratio effectively distinguished between endometriosis and non-endometriosis (139). Notably, resistance to ferroptosis also exists in endometriosis. Liang et al. reported that elevated histone lactylation promotes ferroptosis resistance in ectopic endometrial stromal cells via the HIF1A/heme oxygenase−1 (HMOX1) pathway, regulated by METTL3 (140).

In addition to the metabolic pathways discussed above, emerging evidence suggests that the complement system may act as an upstream regulator of the glycolytic−immune axis. In human macrophages, sublytic concentrations of the membrane attack complex induce an immediate glycolytic shift and collapse of mitochondrial maximal respiration and spare respiratory capacity, accompanied by a time−dependent increase in extracellular lactate (141). This MAC−mediated metabolic reprogramming also activates the NLRP3 inflammasome, leading to the selective release of IL−18 but not IL−1β (142). In the context of endometriosis, clinical evidence further supports complement involvement: peritoneal fluid C3a levels are elevated, C7 is the most overexpressed complement gene in lesions, and decay−accelerating factor (CD55) is significantly reduced, while C3−deficient mice show markedly lower cyst formation (141, 142). Complement activation thus may provide a mechanistic link between metabolic rewiring and immune dysfunction in endometriosis.

### 4.3 Metabolic reprogramming mediates immunosuppression

Accumulating evidence from endometriosis studies suggests that metabolic reprogramming may contributes to immune evasion, although direct causal mechanisms remain incompletely defined (143).

Elevated lactate levels have been detected in the peritoneal fluid of endometriosis patients (144). On the one hand, this lactate−rich environment is hypothesized to directly suppress NK and CTL function, based on cancer models where acidosis reduces CD8^+^ T cell cytolytic activity at pH 6.0–6.5 (145, 146). As shown in cancer models, similar effects are postulated to occur in endometriotic lesions (143). Lactate also downregulates NKG2D receptor expression on NK cells, impairing their cytotoxic capacity (147). Moreover, lactate upregulates PD−L1 on ectopic endometrial cells, engaging PD−1 on T cells to promote immune evasion and T cell exhaustion (143).

On the other hand, lactate promotes M2 macrophage polarization and the accumulation of regulatory T cells. In endometriosis, ascites contain high lactate levels and are associated with a predominance of M2 macrophages (144). Mechanistically, lactate acts via G protein−coupled receptor 81 (GPR81) to activate cAMP−PKA signaling, driving M2 polarization (85). Lactate also enhances Treg infiltration and activity. While extensively studied in tumors, the hyperlactate environment of endometriotic lesions likely fosters Treg−mediated immune suppression (143).

Elevated fatty acid oxidation provides an alternative energy source that fuels cell survival in endometriotic lesions under metabolic stress (128). Lipid metabolites also serve as signaling molecules that regulate immune cell infiltration and activity. Endometriotic cells upregulate prostaglandin E2 synthesis, which suppresses T cell proliferation and NK cell cytotoxicity while promoting M2 macrophage polarization via EP2/EP4 receptors (134). Leukotriene B4, generated during lipid metabolism, recruits neutrophils and monocytes to lesions, where they release cytokines that create a pro−inflammatory milieu and indirectly inhibit adaptive immunity (52).

In endometriosis, tryptophan depletion and kynurenine accumulation in peritoneal fluid have been documented (135, 136). Tryptophan deficiency suppresses T cell proliferation, while kynurenine activates AhR signaling to promote FoxP3^+^ Treg expansion, which in turn inhibits CTL and NK activity (85). Additionally, tryptophan can be produced from tyrosine, which affects the Kynurenine pathway’s activity and encourages cellular immune evasion (148). A Mendelian randomization study by Li et al. suggested a bidirectional causal relationship between tyrosine levels and endometriosis risk. Tyrosine may exacerbate oxidative stress and inflammation, while endometriosis may indirectly elevate tyrosine via metabolic pathways linked to chronic inflammation, liver function, and hormonal balance (149). To increase anticancer activity in immune cells, arginine enhances the proliferation, differentiation, and effector functions of T cells and NK cells (148). Arginine enhances T/NK cell effector functions, but endometriosis exhibits arginine depletion. Chronic stress reduces arginine and serine availability, suppressing T cell activation and promoting lesion spread (150). These findings suggest that multiple amino acid metabolic pathways may contribute to immune evasion in endometriosis.

In EMS, an imbalance in iron homeostasis not only directly triggers oxidative stress by generating abundant ROS but also activates ferroptosis. The production of oxidative stress and ROS is both a consequence and a driver of glycolytic reprogramming (143). Neri et al. reported that ascites-free iron levels are elevated in endometriosis and correlate with an M1/M2 imbalance, with ROS levels directly linked to iron overload (144). By activating the NF-κB signaling pathway, iron excess induces ROS production, which promotes the growth and invasion of ectopic endometrial cells. Concurrently, ROS suppresses the immunological activity of NK and T cells by directly damaging their DNA. Additionally, iron stabilizes HIF-1α, increasing its activity and further enhancing angiogenesis and glycolysis, thereby ensuring lesion survival and progression (143). Elevated ROS directly damages NK and T cell DNA, impairing their immunological activity. Iron excess also triggers ferroptosis via the p53/xCT/GPX4 pathway, which reduces CD8^+^ T cell numbers and compromises granzyme B release, thereby weakening the clearance of ectopic endometrial cells (138, 151). It is important to distinguish two context−dependent roles of ferroptosis in endometriosis. It is important to distinguish two context−dependent roles of ferroptosis in endometriosis. Ferroptosis resistance in ectopic endometrial stromal cells promotes their survival, whereas ferroptosis induction in CD8^+^ T lymphocytes impairs cytotoxic clearance. Thus, resistance enables lesion persistence, while ferroptosis in immune cells facilitates immune escape. These mechanisms are compatible but should be explicitly disambiguated.

**Figure 2:**
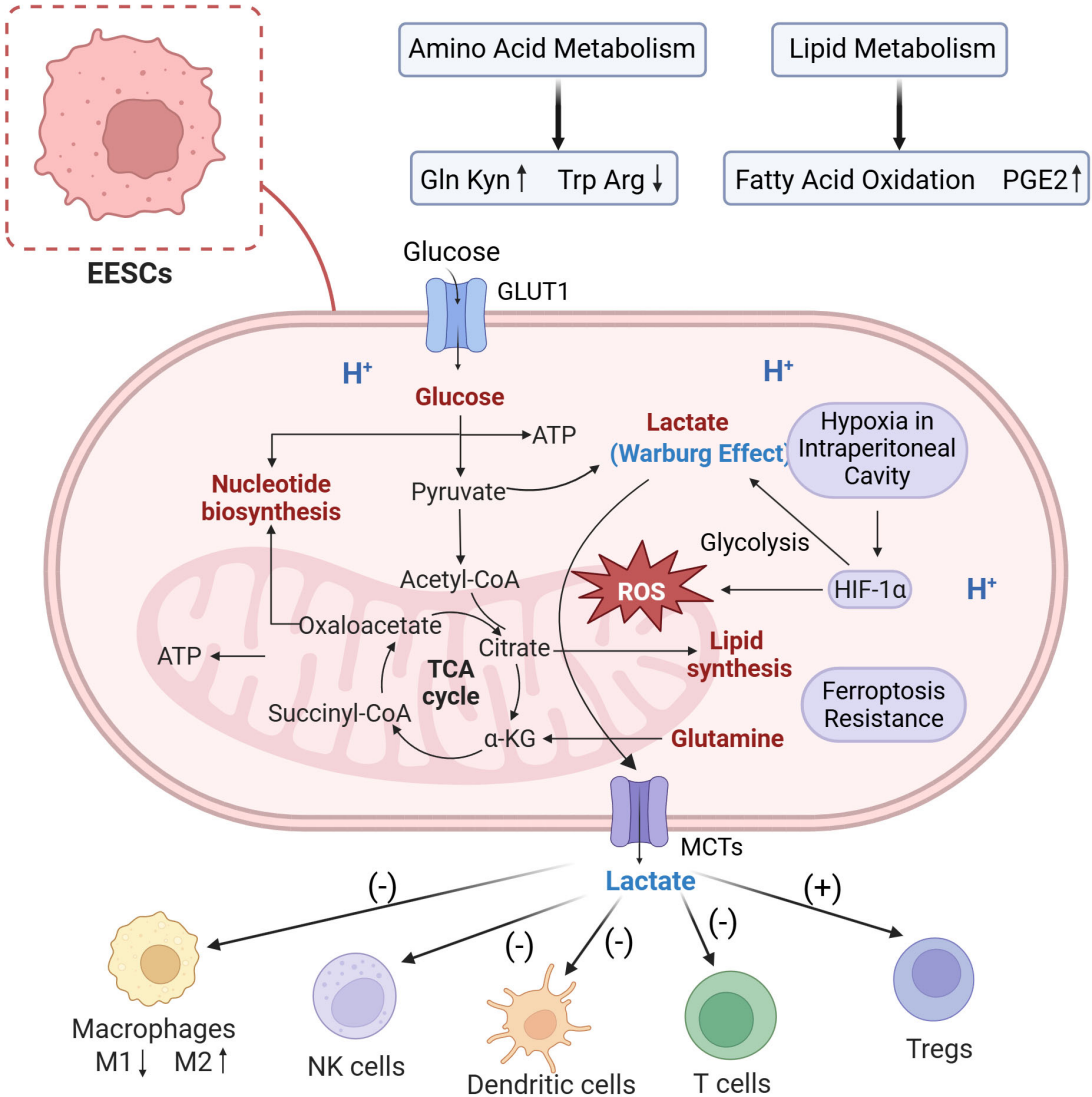
Glycolysis, amino acid, lipid, and iron metabolism microenvironment. Compared with normal endometrial cells, ectopic endometrial cells exhibit unique metabolic alterations, including a shift toward aerobic glycolysis (the Warburg effect), increased fatty acid oxidation, and changes in amino acid and iron metabolism. Hypoxia in the peritoneal cavity activates HIF-1α, which enhances glycolysis and promotes lactate production. This, in turn, fosters an acidic, inflammatory microenvironment that impairs immune cell function, thereby facilitating immune evasion and lesion survival. Enhanced fatty acid oxidation provides an alternative energy source, while alterations in amino acid and iron metabolism further contribute to disease progression. Note: The cell depicted is an endometriosis-associated endometrial stromal cell (EESC). The dashed box encloses the EESC, and the leader line indicates that the central oval is a magnified view of its intracellular details. Regarding the arrows pointing to immune cells: (+) indicates promotion (Treg); (−) indicates suppression (macrophages with M1↓, M2↑, NK cells, T cells, DCs). Created with BioRender.com.

Glycolysis, lipid metabolism, amino acid metabolism, and iron metabolism may collectively contribute to an immunosuppressive milieu in ectopic endometrial cells, which exhibit distinct metabolic traits (143, 144, 151). This metabolic reprogramming may in turn support the development, invasion, and migration of ectopic lesions by potentially facilitating immune evasion (**Figure 2**). In endometriosis, immune evasion appears to be closely associated with metabolic reprogramming. These observations suggest that further experimental research is needed to develop novel metabolic intervention strategies. 

**Figure 3:**
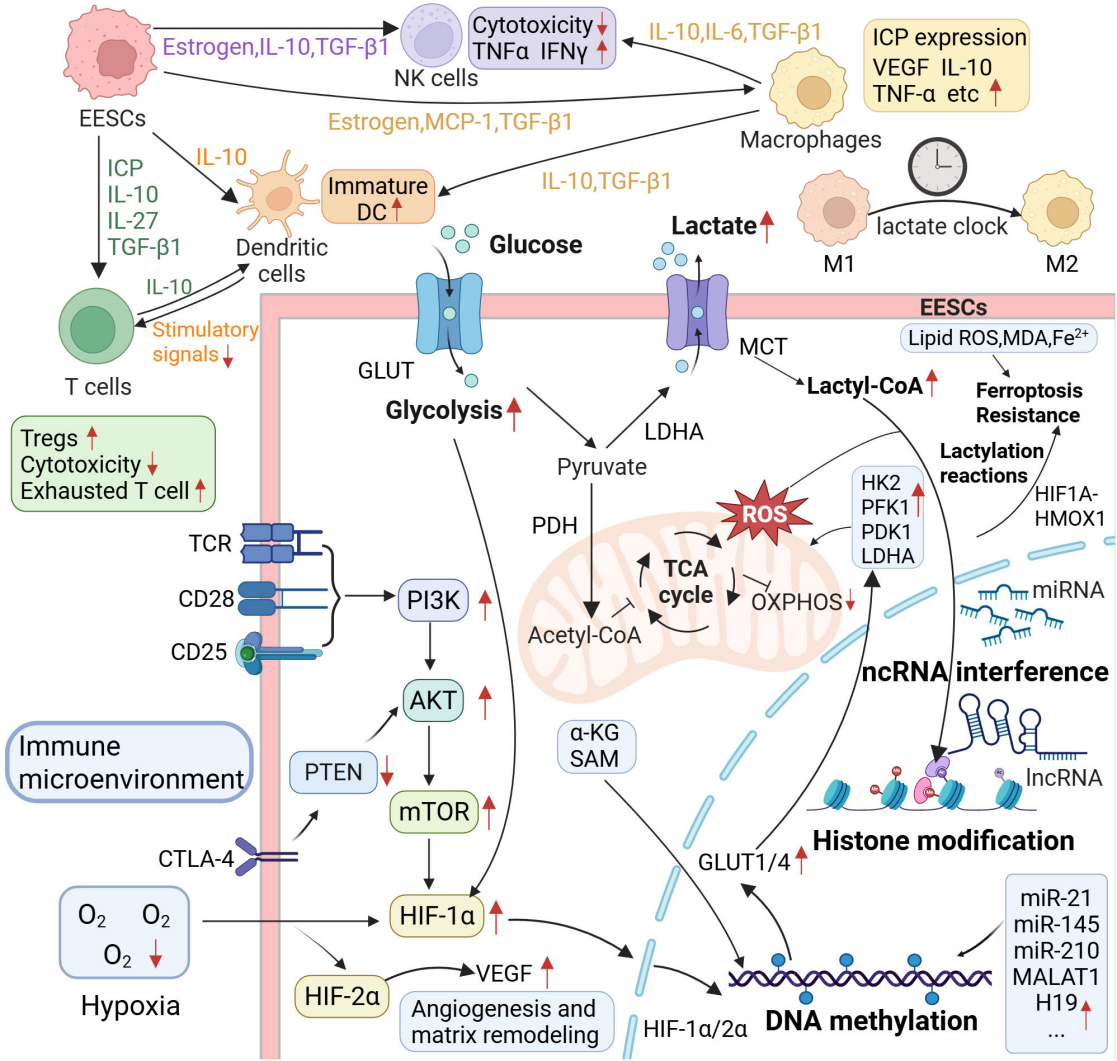
Interacting networks of immunity, epigenetics, and metabolism in endometriosis. The red box represents an ectopic endometrial stromal cell, the primary cell type investigated in endometriosis metabolism and epigenetics research. Within this cell, hypoxia activates HIF-1α, which promotes glycolysis and lactate production. Epigenetic modifications, including DNA methylation, histone modifications, and non-coding RNAs (such as miR-21, miR-145, miR-210, MALAT1, and H19), regulate metabolic mediators and immune-related genes. These changes influence immune cell behavior, including M1/M2 macrophages, regulatory T cells, exhausted T cells, and immature dendritic cells. Collectively, this creates a self-amplifying loop that drives lesion invasion and immune evasion. Created with BioRender.com.

## 5 Interplay of immunity, epigenetics, and metabolism in immune evasion of endometriosis

The evidence reviewed above indicates that metabolic reprogramming, epigenetic alterations, and immune dysfunction converge in endometriosis (14, 20, 118). It is hypothesized that these three domains may form an integrated ‘immune−epigenetic−metabolic axis, ‘ in which positive feedback loops could create and sustain an immunosuppressive microenvironment that supports ectopic lesion survival and growth. If validated, elucidating the interconnections among metabolism, epigenetics, and immunity may inform understanding of disease chronicity and the development of combination therapies.”

### 5.1 Immune signal-induced epigenetic modifications

Signals from immune checkpoint pathways, as well as cytokines, chemokines, and other signaling molecules released by endometrial local immune cells, can directly cause epigenetic changes in ectopic endometrial cells.

Proinflammatory cytokines (TGF−β, TNF−α, IL−1β) activate Smad3 and NF−κB, upregulating DNMT3B, HDAC1/3, and EZH2. For example, TNF−α promotes HDAC3 nuclear translocation via NF−κB. HDAC3 then enhances HK2 transcription and glycolytic dominance, though the precise epigenetic mechanism remains unclear (118, 152). TGF-β regulates DNMT1 via Smad3, suppressing expression of MICA/B—ligands for the NK cell activation receptor NKG2D—thereby diminishing NK cell recognition capacity (118, 153). Furthermore, IL-10 secreted by Treg cells induces EZH2 expression via the IL-10R-STAT3 pathway. EZH2 alters H3K27me3, simultaneously activating PKM2 expression and suppressing IFN-γ transcription, a crucial effector T cell factor (154, 155). Immune checkpoint signaling can also drive epigenetic modification. The binding of PD−L1 on ectopic endometrial cells to PD−1 on T cells upregulates miR−21 via the PI3K/Akt pathway. miR−21 suppresses PTEN, further enhancing PI3K/Akt signaling. Concurrently, miR−21 promotes phosphorylation of histone acetyltransferase p300, accelerating H3K27 acetylation and thereby enhancing transcription of metabolic enzymes and immunosuppressive factors (119, 156).

Therefore, key signaling molecules in the immune microenvironment can directly act as ‘epigenetic editors’, reshaping the functional states of immune cells by regulating DNA methylation, histone modification, and non-coding RNA.

### 5.2 Metabolite-mediated epigenetic regulation

Metabolites generated by metabolic reprogramming of ectopic endometrial cells can act as co−substrates, inhibitors, or signaling molecules for epigenetic modifiers, and may influence immune−related gene expression (85, 143). Changes in intracellular metabolism alter levels of key metabolites, including acetyl−CoA, α−KG, and S−adenosylmethionine (SAM), thereby modifying histone acetylation, DNA methylation, and histone methylation (157). For example, elevated glycolysis and fatty acid oxidation increase acetyl−CoA availability. In cancer models, acetyl−CoA serves as a substrate for histone acetyltransferases (e.g., p300/CBP), promoting H3K27 acetylation and transcriptional activation (158). Whether this occurs in endometriosis remains unknown. The glutamine−derived metabolite α−KG promotes demethylation by acting as a cofactor for TET DNA demethylases and JmjC histone demethylases, whereas succinate competitively inhibits these enzymes, leading to DNA and histone hypermethylation (159). In tumor microenvironments, an altered α−KG/succinate ratio can establish an inhibitory epigenetic landscape. Given that lactate and succinate accumulate in endometriosis, it is hypothesized that similar mechanisms may contribute to immune tolerance, but direct evidence is lacking (143, 159). Methionine deficiency reduces SAM levels, a cofactor for histone methyltransferases, decreasing histone H3 di− and trimethylation and affecting T cell effector gene expression (85, 160). These findings derive primarily from T cell biology; their relevance to endometriosis−associated immune cells requires direct investigation.

Reactive oxygen species (ROS) also mediate metabolite−epigenetic crosstalk. ROS affect epigenetic enzymes (DNMTs, HDACs, HMTs) via redox−sensitive modifications (85). Lactic acid and pyruvate can induce ROS generation in a dose−dependent manner, as shown in cellular models; at critical thresholds, ROS drives oxidative damage, transcriptional dysregulation, and subsequent epigenetic remodeling (85). Whether this cascade operates in endometriotic lesions is plausible but has not been directly demonstrated. Collectively, the metabolic−epigenetic crosstalk outlined above is well established in cancer and immunology but has received limited direct validation in endometriosis. Future studies should prioritize metabolomic and epigenomic profiling of endometriosis lesions and lesion−infiltrating immune cells to test these hypotheses.

### 5.3 Epigenetic regulation of immune metabolic reprogramming

Epigenetic modifications are increasingly recognized as an important link between metabolism and immune function (161, 162). These changes can influence immunometabolic programming and sustain disease−associated phenotypes by regulating the transcription of key genes that encode both metabolic enzymes and immune molecules. However, it is important to note that most of these mechanistic insights are currently derived from cancer and general immunology studies, with direct experimental validation in endometriosis remaining limited (134, 157).

In the context of basic immunology, DNA methylation is a key regulator governing CD4^+^ T cell lineage commitment and cytokine production. For instance, TGF−β is known to upregulate FOXP3 expression, an event associated with the hypomethylation of intronic CpG islands, whereas the inhibition of TET1/2 can block CNS2 demethylation, thereby suppressing FOXP3 expression and impairing Treg differentiation (161). The functional stability of Tregs is known to be determined by the demethylation status of enhancer regions such as CNS2 within the FOXP3 gene. Concurrently, the inhibition of p300/CBP has been shown to reduce H3K18ac and H3K27ac levels, leading to the downregulation of Foxp3 and its protein deacetylation in Tregs (163). DNMTs also play a vital role in the lineage development, survival, and expansion of CD8^+^ T lymphocytes (161). Furthermore, DNA methylation is essential for the differentiation and cytotoxic function of NK cells, and histone modifications modulate cytokine release by these cells. Various histone methyltransferases can deposit activating (e.g., H3K4me3) or repressive (e.g., H3K9me2, H3K27me2) marks at the promoters of genes such as TNF, IL6, and TNFAIP3, thereby playing a key role in influencing macrophage M1/M2 polarization (164).

A link between epigenetics and immune cell metabolic preferences has been suggested (157). In M2 macrophages and Tregs, genes involved in oxidative phosphorylation and fatty acid oxidation display activating histone marks, such as H3K4me3. In contrast, glycolytic genes in these cells are often enriched with repressive marks such as H3K27me3, a pattern consistent with their lineage−specific metabolic programming (161, 162). This suggests that cytokine−driven or microenvironment−induced epigenetic programs can establish distinct metabolic states in immune cells to support their specialized effector functions. The reversible nature of these epigenetic modifications makes them promising targets for therapeutic strategies (134, 157).

Recent studies provide direct evidence of this epigenetic−immune−metabolic crosstalk in endometriosis. For example, iron overload in endometriotic lesions has been shown to drive ferroptosis resistance via the METTL3−HIF1A−HMOX1 pathway, which in turn promotes M2 anti−inflammatory macrophage polarization. These M2 macrophages are known to express CCL2, CSF1, and IL−6, which can reinforce local immune tolerance and disrupt the critical Th17/Treg balance (151). Furthermore, elevated levels of histone lactylation (e.g., H3K18lac), which directly links glycolytic lactate production to epigenetic modification, have been found to enhance ferroptosis resistance in ectopic endometrial stromal cells by facilitating the clearance of lipid peroxides, thereby supporting their survival and proliferation (140, 157). Collectively, these findings offer initial evidence that epigenetics may serve as an integrative node connecting metabolism and immunity in endometriosis, although the full extent and precise mechanisms of this axis remain to be fully elucidated (134).

### 5.4 Direct crosstalk between immunity and metabolism

Direct metabolic crosstalk between immune cells and ectopic endometrial cells, which operates independently of epigenetic modifications, is thought to contribute to a self−amplifying immunosuppressive loop that promotes endometriosis progression. This crosstalk can occur through two interrelated mechanisms (143, 144). Metabolic products exchanged between cells can act as signaling molecules to regulate immune cell function, and shared intracellular signaling pathways may simultaneously modulate inflammation and metabolism. Hypoxia, ROS, iron overload, and lipid metabolic disorders in the endometriotic microenvironment may collectively remodel immune cell metabolism, altering their effector functions and polarization states (143, 148).

The effects of specific metabolites on immune cells are as follows. Lactic acid accumulation inhibits PFK1, a key glycolytic enzyme in effector T cells, thereby impairing their proliferation. In contrast, Tregs rely more on oxidative phosphorylation. High concentrations of glutamine support Treg survival and functional maintenance (165). ROS directly damage NK cell DNA, downregulate NKG2D receptor expression, and suppress cytotoxic activity (143). PGE2 reduces macrophage phagocytosis by downregulating scavenger receptors and the lipid transporter CD36, and increases endometriotic lesion size in mice (134). Abnormal cholesterol ester accumulation in ectopic lesions upregulates COX−2 and PGE2, creating a self−reinforcing inflammatory cycle that drives innate immune dysfunction and chronic inflammation (85). Beyond these metabolite−specific effects, shared NF−κB and PI3K/Akt/mTOR pathways in both immune cells and lesions concurrently upregulate inflammatory and immunosuppressive factors (for example, IL−6, IL−10, COX−2 and PGE2) and metabolic enzymes (for example, HK2 and lipid synthesis genes), perpetuating the pathogenic microenvironment (85, 166).

### 5.5 A reciprocal regulatory loop connecting immunity, epigenetics, and metabolism

Before presenting our proposed axis, it is important to position it against existing multi−dimensional frameworks. The ImmunoMET Oncogenesis model links inflammation, metabolism, and proliferation as interdependent drivers of malignant transformation (167). The trained immunity paradigm explicitly couples epigenetic reprogramming with metabolic reprogramming in innate immune cells to produce sustained inflammatory memory (168). The microbiota−metabolism−epigenetics−immunity axis demonstrates a concrete mechanistic pathway: Lactobacillus johnsonii−derived indole−3−propionic acid enhances CD8^+^ T cell stemness via H3K27 hyperacetylation at the Tcf7 super−enhancer region, thereby enhancing anti−tumor immunity (169). These frameworks, while highly informative, are either cancer−centric, focused on immune cell−intrinsic memory, or centered on gut−derived signals. To our knowledge, it remains unclear whether these frameworks can be directly applied to the bidirectional crosstalk among ectopic endometrial cells, their metabolic state, and local immune dysfunction in endometriosis.

In contrast, our analysis hypothesizes metabolic reprogramming as a potential initial driver, epigenetic modification as a key transducer, and immune dysfunction as the final output, which may collectively form a dynamic closed loop (the “metabolism−epigenetics−immunity” axis) that could maintain the immunosuppressive microenvironment of ectopic lesions (**Table 2**). We explicitly acknowledge that the proposed directionality has not been established by longitudinal human studies and therefore represents a working hypothesis requiring experimental validation. Thus, our axis is not claimed as an established mechanism but rather as a testable framework tailored specifically to endometriosis, offering a perspective that may complement existing models.

**Table 2:** The interplay of immune, epigenetic, and metabolic mechanisms in endometriosis immune evasion.

Regulatory axis	Metabolic alteration	Epigenetic mechanism	Immune evasion effect	References
Glycolysis-Epigenetic-Immune Axis	Enhanced Warburg effect; lactate accumulation	Histone lactylation (H3K18lac)	Suppresses T/NK cytotoxicity; promotes M2/Treg polarization; supports angiogenesis and fibrosis	(125, 140, 145, 146)
Lipid Metabolism - Epigenetic-Immune Axis	Increased PGE2 synthesis; lipid peroxidation	Metabolites act as cofactors/ligands for epigenetic enzymes	Self−reinforcing PGE2 loop; disrupts immune cell function; promotes chronic inflammation and fibrosis	(118, 130, 133, 158)
Amino Acid Metabolism-Epigenetic-Immune Axis	Tryptophan depletion with kynurenine accumulation. Arginine depletion. Glutamine elevation.	SAM, α−KG, succinate serve as cofactors/substrates for epigenetic enzymes	Suppresses NK/effector T cells; expands Tregs/M2 macrophages; promotes lesion fibrosis and adhesion	(140, 148, 150, 170)
Iron Metabolism - Epigenetic-Immune Axis	Iron overload; ROS generation via Fenton reaction	ROS−mediated oxidative stress; histone lactylation; METTL3−HIF1A−HMOX1 pathway	Ferroptosis resistance (lipid peroxide scavenging); promotes cell survival and proliferation	(139, 140, 151)

The “glycolysis-epigenetic-immune axis” is a central driver of the complex network of immune evasion in endometriosis (**Figure 3**). Endometriotic lesions are characterized by hypoxia, driven by the peritoneal environment, poor vascularization, and high metabolic demands (143). The hypoxic microenvironment maintains HIF−1α, which drives aerobic glycolysis in immune cells (e.g., macrophages, Tregs) and upregulates survival factors while inhibiting oxidative phosphorylation – mechanisms detailed in Section 4.1 for lesion cells, and similarly proposed for immune cells. McKinnon et al. reported that this activation increases glucose transporters (GLUT1, GLUT4), ensuring a sustained glucose supply (171). In addition to fueling cell division, this metabolic shift generates key intermediates such as α-KG and acetyl-CoA. α-KG is a necessary cofactor for TET DNA demethylases and JmjC histone demethylases, initiating DNA demethylation, while acetyl-CoA serves as a substrate for histone acetylation (85). Glycolysis in endometriosis is further influenced by genetic and epigenetic factors. DNA methylation, histone modifications, and microRNAs (miR-21, miR-145, miR-210) affect mitochondrial and metabolic pathways (143, 172). Key lncRNAs, including H19, promote glycolytic enzyme expression and influence glucose metabolism in endometriosis. Wen et al. found that elevated H19 drives abnormal glucose metabolism, leading to lactate accumulation—a substrate for histone lactylation. This may represent a key mechanism by which H19 enhances cell proliferation and migration, accelerating endometriosis progression, as demonstrated in a mouse model (173). Histone lactylation enables lactate to modify histone lysine residues directly, thereby altering gene transcription (140, 174). The PI3K/AKT pathway, essential for endometrial stromal cell survival and proliferation, is also epigenetically regulated by histone modifications and microRNAs (85).

Ultimately, these changes intensify the Warburg effect, raising lactate concentrations and glycolytic rates and generating an acidic milieu that promotes inflammation and immune evasion. Accumulated lactate, imported via MCT1, induces IκBα phosphorylation and activates the NLRP3 inflammasome and NF-κB pathways, driving inflammation (85). Lactate also activates GPR81, increasing CXCL1 and CXCL2 production, which recruits and activates neutrophils and macrophages. Enhanced glycolysis fuels macrophage polarization by providing ATP and lactate as a carbon source. Over time, lactate accumulation shifts macrophages toward an M2-like anti-inflammatory phenotype (174, 175). This phenotypic shift creates an immunosuppressive milieu in endometriosis, with TGF−β and IL−10 secretion helping ectopic cells evade immune surveillance. High lactate impairs NK cell effector function, reducing IFN−γ and TNF−α production and inducing NK cell apoptosis. Elevated lactate also expands MDSCs, indirectly suppressing NK and T cell activity (176). Lactate promotes Treg differentiation, contributing to inflammation resolution. Tregs, metabolically distinct from effector T cells, take up lactate, convert it to pyruvate, and then to citrate and malate to fuel the TCA cycle; malate is further converted to oxaloacetate and phosphoenolpyruvate to support gluconeogenesis, ultimately promoting Treg proliferation and immune suppression (177, 178). Furthermore, lactate acts as an HDAC inhibitor, leading to increased H3K27 acetylation. This enhances the stem-like phenotype of CD8+ T cells and promotes Treg differentiation and function. Lactate also suppresses CD8+ T cell proliferation, cytokine production, and cytotoxicity by inhibiting NFAT and shifting pyruvate utilization (177).

In response to microenvironmental stressors including pelvic hypoxia and estrogen stimulation, ectopic endometrial cells may undergo metabolic reprogramming during an early adaptive phase, potentially increasing lipid synthesis and glycolysis to support lesion survival (144). During a subsequent signal transduction phase, metabolic byproducts such as lactate, acetyl-CoA, and α-KG could influence epigenetic enzyme activity, while cytokines secreted by immune cells (e.g., TGF-β, IL-10) may induce epigenetic remodeling that in turn affects transcription of immunosuppressive molecules and metabolic enzymes (14). In a phenotypic output phase, metabolic reprogramming may contribute to lesion invasion and proliferation, and immunosuppressive molecules (e.g., PD-L1, IDO) together with immunosuppressive cells (e.g., M2 macrophages, Tregs) may facilitate immune evasion and lesion persistence (179). Over time, ongoing immune cell infiltration and cytokine release could further promote metabolic and epigenetic alterations, potentially creating a self-reinforcing loop (118). This framework may help to explain some clinical features of endometriosis, including its chronicity and tendency to recur.

It is important to emphasize that many of the mechanistic links described above are extrapolated from oncology and immunology literature. Direct experimental evidence demonstrating these interconnected processes in human endometriosis remains limited. Different lesion subtypes (peritoneal, ovarian, deep infiltrating) may exhibit distinct immune, metabolic, and epigenetic profiles (18). Moreover, clinical parameters such as pain severity, infertility, and lesion burden are often poorly correlated with one another, and no reliable biological measure has yet been shown to directly predict lesion behavior or patient experience (52). Therefore, the proposed “immune-epigenetic-metabolic” axis may provide one possible framework for understanding selected aspects of endometriosis biology, but it may not operate uniformly across all lesion types and patient populations.

## 6 Conclusion

Current literature indicates that immune dysfunction, epigenetic alterations, and metabolic reprogramming each contribute to endometriosis pathogenesis. However, whether these three layers interact in a causal, self−amplifying manner remains unknown. The “Immune−Epigenetic−Metabolic Axis” proposed in this review is a conceptual model that integrates existing observations; it has not been directly validated in human endometriosis. Several limitations must be acknowledged. Endometriotic lesions exhibit substantial heterogeneity across peritoneal, ovarian and deep infiltrating subtypes, and these subtypes may not share identical immune, metabolic or epigenetic profiles. Clinical symptoms such as pain and infertility correlate poorly with lesion burden or molecular features, and no reliable biological measure of disease severity currently exists. Most mechanistic insights discussed in this review are derived from cancer and immunology studies; direct experimental evidence from human endometriosis remains limited. To test the hypothesis that an integrated axis exists, future research should prioritize several complementary approaches. Single−cell multi−omics (transcriptomics, epigenomics and metabolomics) applied to paired lesions and immune cells from well−phenotyped patients could identify cell−type−specific signatures. Functional animal models that allow temporal dissection of the axis, such as inducible metabolic gene knockouts or pharmacological interventions after lesion establishment, would help establish causal directionality. Prospective clinical cohorts correlating metabolic and immune markers with disease outcomes are also needed. Only after such validation can the proposed axis inform rational combination therapies. For now, it remains a testable framework, not an established mechanism.

## Glossary

EMS: Endometriosis
NK: Natural killer
NKG2D: Natural killer group 2 member D
CTLs: Cytotoxic T lymphocytes
IL: Interleukin
TGF: Transforming growth factor
Tregs: Regulatory T cells
TAMs: Tumor-associated macrophages
JAK: Janus kinase
STAT: Signal transducers and activators of transcription
ESCs: Endometrial stromal cells
DCs: Dendritic cells
ICPs: Immune checkpoints
PD-1: Programmed cell death receptor-1
PD-L1: Programmed death-ligand 1
IFN: Interferon
TIM-3: T cell membrane protein-3
Gal-9: Galectin-9
CTLA-4: cytotoxic T lymphocyte-associated antigen-4
PB: Peripheral blood
PF: Peritoneal fluid
IDO: 2, 3-dioxygenase
TNF: Tumor necrosis factor
DNMTs: DNA methyltransferases
Cf-DNA: Cell-free DNA
HDACs: Histone deacetylases
HATs: Histone acetyltransferases
HMTs: Histone methyltransferases
PGE2: Prostaglandin E2
PFK: Phosphofructokinase
HK2: Hexokinase 2
PDK1: Phosphoinositide-dependent protein kinase 1
AKT: Protein kinase B
mTOR: Mammalian target of rapamycin
PTEN: Phosphoinositide 3-kinase activator
HIF-1α: Hypoxia-inducible factor-1α
GLUT: Glucose transporter
α-KG: α-ketoglutarate
SAM: S-adenosylmethionine
TCA: Tricarboxylic acid cycle
HMOX1: HIF1A/heme oxygenase 1
METTL3: Methyltransferase-like 3
ROS: Reactive oxygen species
